# Supplementary material for: Surface Transformation of Spin-on-Carbon Film via Forming Carbon Iron Complex for Remarkably Enhanced Polishing Rate
Source: Nanomaterials (Basel). 2022 Mar 15;12(6):969. doi: 10.3390/nano12060969 (PMC8953943; doi:10.3390/nano12060969)
Supplement: Supplementary file 1 [file nanomaterials-12-00969-s001.zip › nanomaterials-1620634-supplementary.pdf]

Supplementary Materials

Surface Transformation of Spin-On-Carbon Film via Forming Carbon Iron Complex for Remarkably Enhanced Polishing Rate

Jun-Myeong Lee [1], Jong-Chan Lee [1], Seong-In Kim [1], Seung-Jae Lee [2], Jae-Yung Bae [3], Jin-Hyung Park [4] and Jea-Gun Park [1,2,*]

[1] Department of Nanoscale Semiconductor Engineering, Hanyang University, Seoul 04763, Korea; kjunom200@naver.com (J.-M.L.); student.jongchan@gmail.com (J.-C.L.); rlatjddls25805@naver.com (S.-I.K.)
[2] Department of Electronic Engineering, Hanyang University, Seoul 04763, Korea; ltwobooml@naver.com
[3] Department of Energy Engineering, Hanyang University, Seoul 04763, Korea; jaeyoungb@daum.net
[4] UB Materials Inc., Yongin 17162, Korea; parkjinhyung@gmail.com
* Correspondence: parkjgl@hanyang.ac.kr

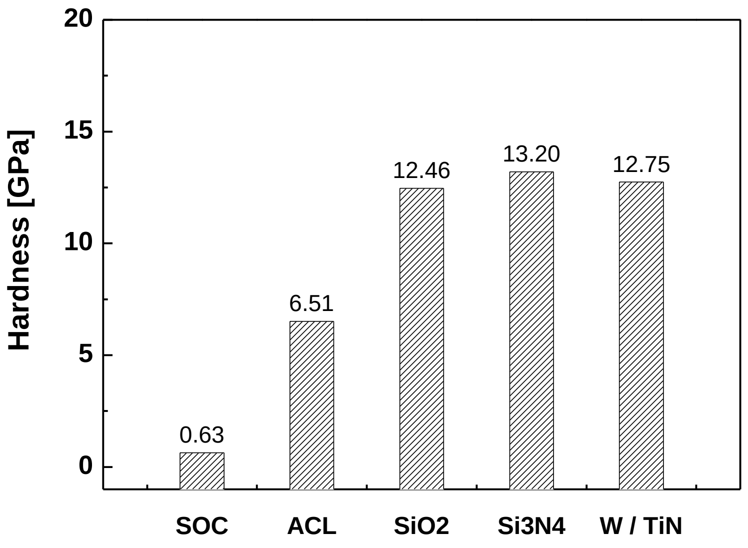

Figure S1. Dependencies of hardness on the film types. SOC and ACL are spin-on-carbon and amorphous carbon layer.

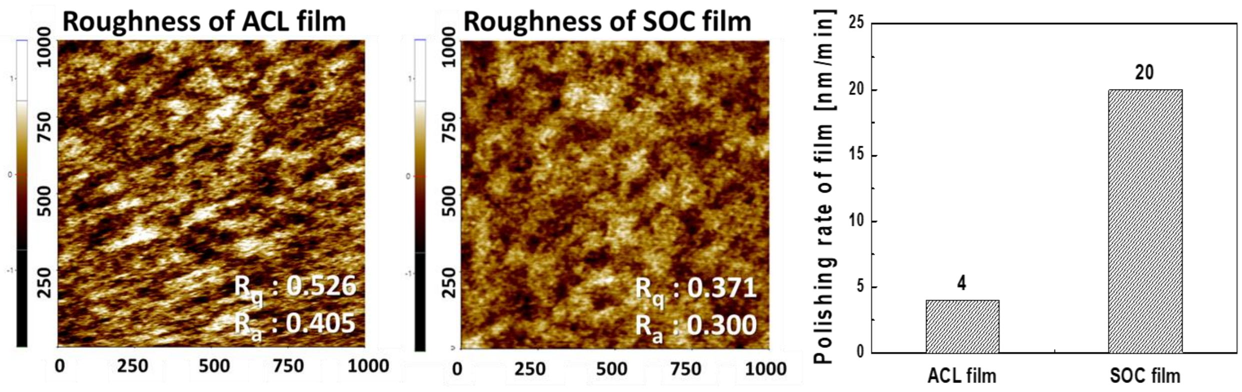

Figure S2. Roughness of the SOC and ACL film surface by using AFM (1umx1um in scanning area).

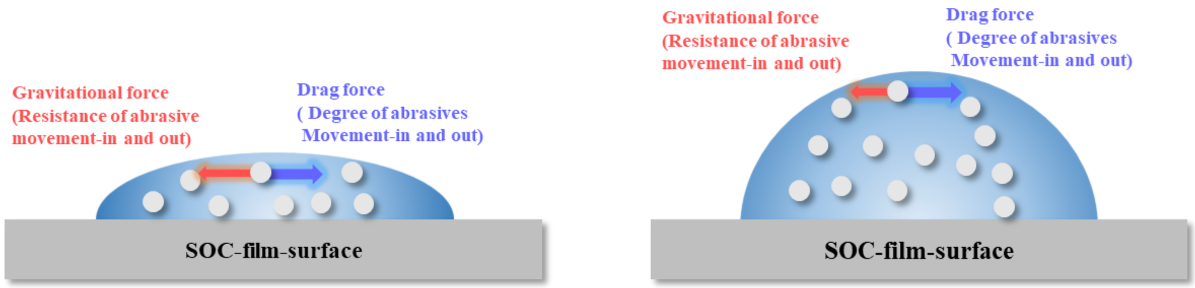

Figure S3. Stoke's law for the abrasive movement in the CMP slurry. The drag force (F_D) due to the resistance of fluid and gravitational force(F_m) are defined by the equations (1) and (2).

$$F_D = C_D A \rho_f (\frac{v^2}{2}) = 3\pi d_d \eta v \qquad (1)$$

$$F_m = \frac{\pi}{6} d^3 (\rho_s - \rho_f) g = \frac{\pi}{6} d_v^3 (\rho_s - \rho_f) g \qquad (2)$$

C_D, A, ρ_f, d_d, η, ρ_s, and g are the drag coefficient depending on abrasive size, shape, velocity, the projected area of a abrasive perpendicular to direction of motion, the fluid density relative velocity of an abrasive in a fluid, the drag diameter, the fluid viscosity, the density of an abrasive, and the gravitational acceleration, respectively. Since A decreases with decreasing the contact angle (i.e., absorption degree: hydrophilicity) of the SOC-film-surface CMP slurry on the polished SOC-film-surface, F_D decreased with increasing the ferric catalyst concentration, resulting in a less the movement out and in of the ZrO_2 abrasives on the CMP pad.

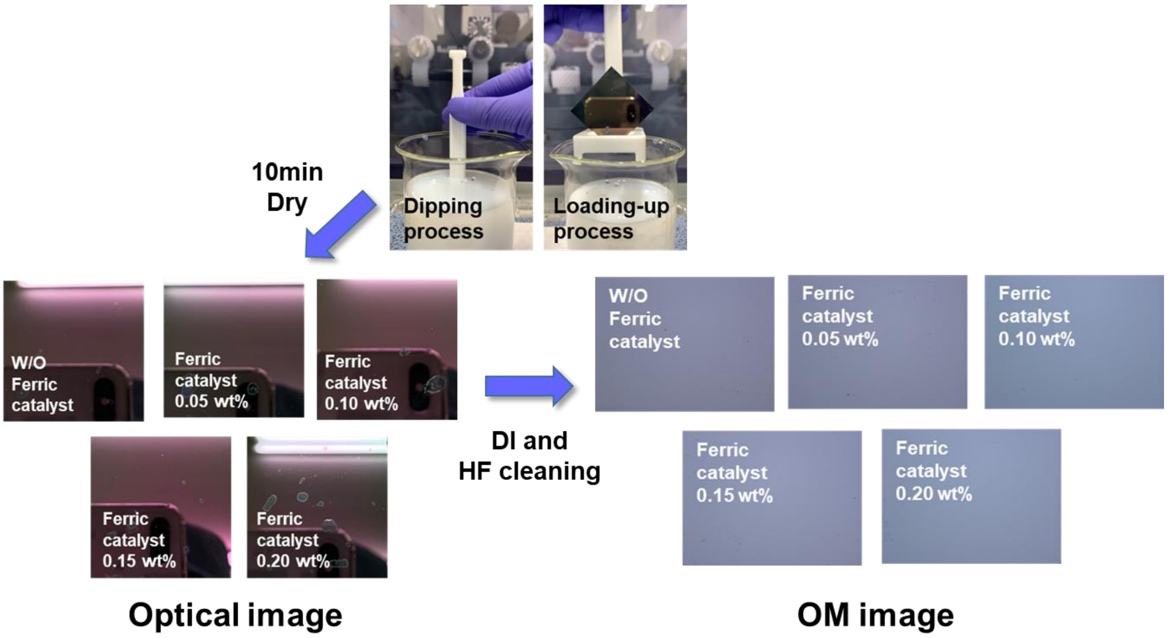

Figure S4. Adsorption degree (i.e., image of the SOC film after dipping and loading-up process and DI and HF cleaning) of the SOC film surface CMP on SOC film surface, depending on the ferric catalyst concentration.

Table S1. SOC film polishing rate depending on the types of iron-based catalysts.

List	Dissociated chemical species	Initial pH @1wt% solution	$Fe(OH)_x$ precipitation @pH 2.3	SOC Polishing rate @3psi,30s
Ammonium iron(Ⅲ) citrate $(NH_4)_5[Fe(C_6H_4O_7)_2]$	2[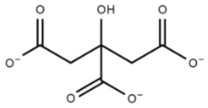]$^{3-}$, Fe^{3+}, $5NH_4^+$	5.94	X	829
Ammonium iron(Ⅲ) oxalate trihydrate $(NH_4)_3[Fe(C_2O_4)_3]\cdot 3H_2O$	3[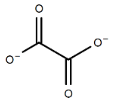]$^{2-}$, Fe^{3+}, $3NH_4^+$, $3H_2O$	5.32	X	787
Iron(Ⅲ) sulfate hydrate $Fe_2(SO_4)_3\cdot H_2O$	3[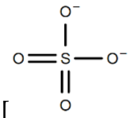]$^{2-}$, $2Fe^{3+}$, H_2O	2.63	X	2022
Propylenediamine tetra-acetic acid Iron(III) $C_{11}H_{18}N_2O_8$	3[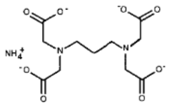]$^{4-}$, $4Fe^{3+}$, H_2O	2.78	X	1814
Iron(Ⅲ) nitrate enneahydrate $Fe(NO_3)_3\cdot 9H_2O$	3[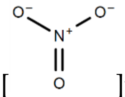]$^{-}$, Fe^{3+}, $9H_2O$	2.43	X	1211
Potassium ferrocyanide $K_4Fe(CN)_6$	$4K^+$, [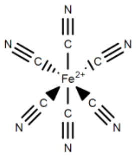]$^{4-}$	6.81	X	808

*ALL SOC CMP slurries were titrated a pH of 2.3 by adding the titrant (i.e., HNO_3)
